# Diverse DTPA-chelated lanthanides with relevance for nuclear medicine bound to an engineered lipocalin reveal conserved ligand geometry

**DOI:** 10.1107/S2053230X26007624

**Published:** 2026-08-17

**Authors:** Alina Mirwald, Andreas Eichinger, Arne Skerra

**Affiliations:** a School of Life Sciences Technical University of Munich 85354Freising Germany; The Scripps Research Institute, USA

**Keywords:** Anticalins, lipocalins, metal-chelate complexes, protein engineering, X-ray crystallography

## Abstract

The Anticalin CL31d, an engineered lipocalin protein previously designed to specifically bind rare-earth and related metal ions as chelate complexes with *p*-NH_2_-Bn-CHX-A″-DTPA (DTPA-NH_2_), was subjected to structural and binding studies with a series of 11 different *M*^III^·DTPA-NH_2_ complexes. These complexes include various lanthanide and main-group metal(III) ions whose radioisotopes are useful in nuclear medicine, with ionic radii ranging from 0.62 to 1.03 Å. Binding activities of the Anticalin for the *M*^III^·DTPA-NH_2_ complexes were quantified by fluorescence titration (probing the intrinsic Tyr/Trp emission), revealing *K*_d_ values of 0.8–2.4 n*M* for most of the lanthanide ions investigated (*r*_ion_ = 0.75–0.96 Å), but showing markedly reduced affinities towards the small and large main-group metal ions Ga^3+^ (*K*_d_ = 15.3 nM) and Bi^3+^ (*K*_d_ = 47.8 n*M*), respectively. The crystal structures of six representative *M*^III^·DTPA-NH_2_ complexes bound to the Anticalin were solved at high resolution (1.5–1.8 Å) using synchrotron X-ray diffraction. Superposition onto the previously described Anticalin CL31 with bound Y^3+^·DTPA-SCN indicated an essentially invariant conformation both for the binding protein and its metal-chelate ligands, including conserved hydrogen bonds, and a surprisingly uniform ninefold metal coordination via five carboxylate groups, three N atoms and one water molecule. However, there were two exceptions: the small Sc^3+^ ion appeared to be coordinated only eightfold with the DTPA-NH_2_ chelator but lacking the water ligand, whereas no metal electron density was observed for the Ga^3+^ ion, in line with its known noncanonical DTPA complex geometry. Interestingly, in this case a water molecule was detected at the expected position of the central metal ion within the protein-bound DTPA-NH_2_ chelator. Our investigation of the influence of chelate geometry on complex stability establishes the Anticalin CL31d as a small and robust universal binding protein for medically relevant *M*^III^·DTPA complexes with surprisingly broad tolerance towards varying ionic radii, thus enabling flexible radionuclide-targeting strategies in nuclear medicine.

## Abstract 2

Structural and binding studies of the engineered lipocalin CL31d towards the chelate complexes of 11 different lanthanide and main-group metal(III) ions, whose radioisotopes are useful for nuclear medicine, with a functionalized DTPA revealed a surprisingly broad tolerance towards varying ionic radii, thus enabling flexible radionuclide-targeting strategies.

## 1. Introduction

Antibodies offer huge potential for both *in vivo* diagnostics and tumor therapy, nowadays referred to as theranostics (Burkett *et al.*, 2023 ▸), due to their high specificity and affinity towards disease-relevant molecular targets (Goldenberg & Sharkey, 2006 ▸; Milenic *et al.*, 2004 ▸). However, their large size limits tissue penetration and leads to prolonged circulation, which can hamper tumor-imaging applications via positron emission tomography (PET) or single-photon emission computed tomography (SPECT) (Boswell & Brechbiel, 2007 ▸; Löfblom *et al.*, 2011 ▸). Although antibody fragments such as Fab or scFv, as well as single-domain immunoglobulins (Igs) from camels or sharks, alleviate some of these limitations (Holliger & Hudson, 2005 ▸), there remains considerable interest in alternative non-Ig protein scaffolds that offer comparable high-affinity and specific target recognition. Over the past two decades, more than 50 such non-Ig binding proteins with engineered ligand specificities have been described (Gebauer & Skerra, 2019 ▸), with a subset already investigated for PET and SPECT imaging applications. For conventional tumor imaging, both antibody-derived and non-Ig binding proteins are chemically conjugated to a radionuclide, potentially mediated by a metal chelator, and employed to target a disease-related antigen.

Alternatively, such binding proteins may serve to fix the radionuclide by way of biomolecular recognition and non­covalent complex formation, for example as part of a bispecific antibody or fusion protein. The potential of an antibody-based system for the tight binding of complexes between rare-earth metal ions and the chelator 1,4,7,10-tetraazacyclo­dodecane-*N*,*N*′*N*′′,*N*′′′-tetraacetic acid (DOTA) in biomedical applications was demonstrated with the Fab fragment of the monoclonal antibody (MAb) 2D12.5, which was originally raised against a Y^3+^·DOTA analog (Corneillie, Whetstone *et al.*, 2003 ▸). This Fab showed high affinity towards *M*^III^·DOTA complexes across the entire lanthanide series, often comparable to, or even better than, that of the original complex with Y^3+^, as measured by a competitive immunoassay. Of note, while naked lanthanide ions tend to form precipitates under physiological or weakly alkaline pH conditions, or may unspecifically interact with biomolecules, their DOTA complexes are inert and remain highly soluble even under *in vivo* conditions. However, the precise geometry of metal complexes with this cyclic chelator varies depending on their ionic radius. Indeed, the binding strength between the 2D12.5 Fab and the series of lanthanide ions in complex with DOTA showed a parabolic correlation with optimal affinity near Y^3+^, corresponding to an effective ionic radius *r*_ion_ ≃ 1.075 Å (for coordination number CN = 9; Shannon, 1976 ▸), and decreasing affinity for larger or smaller ions. This is likely due to changes in the metal-chelate geometry, although X-ray crystallo­graphic analysis of the 2D12.5 Fab bound to DOTA analogs in complex with either Y^3+^ or Gd^3+^, two metal ions with very similar ionic radii, indicated only minute structural deviations (Corneillie, Fisher *et al.*, 2003 ▸).

Compared with Fab fragments of antibodies, engineered non-Ig scaffolds, such as Anticalins, which are derived from human lipocalin proteins, offer a smaller size and stable single-chain protein fold as well as improved tissue penetration while enabling high-affinity metal-chelate recognition, which makes them promising alternatives for PET or SPECT imaging. In fact, human lipocalin 2 (Lcn2; also known as neutrophil-gelatinase-associated lipocalin, NGAL), an abundant plasma protein, naturally binds iron-chelate complexes, so-called siderophores (Goetz *et al.*, 2002 ▸). Furthermore, engineered versions of Lcn2 have been developed, dubbed Anticalins, which specifically bind Fe^3+^ or Ga^3+^ in complex with the bacterial chelators petrobactin (Dauner *et al.*, 2018 ▸) as well as pyoverdine and pyochelin (Dauner & Skerra, 2020 ▸). Other Anticalins have been engineered to recognize lanthanide complexes with derivatives of diethylenetriaminepentaacetic acid (DTPA; Eggenstein *et al.*, 2014 ▸; Kim *et al.*, 2009 ▸), an acyclic chelator that is frequently used for the preparation of radionuclide conjugates in nuclear medicine (Brechbiel & Gansow, 1991 ▸, 1992 ▸).

Generally, lipocalins are small, soluble secretory proteins found in many organisms, including humans, where they are involved in the transport or storage of a wide range of biomolecules (Schiefner & Skerra, 2015 ▸; Skerra, 2000 ▸). The structural core of lipocalins is highly conserved, featuring a β-barrel formed by eight antiparallel strands with four structurally variable loops at its open end, which can be reshaped to specifically bind novel targets (Beste *et al.*, 1999 ▸). This can be achieved by generating random libraries via targeted mutagenesis and combinatorial selection, resulting in so-called Anticalins, which leverage the stable and versatile lipocalin scaffold to create alternative binding proteins for diagnostic and therapeutic applications (Achatz *et al.*, 2022 ▸; Deuschle *et al.*, 2021 ▸; Gebauer & Skerra, 2012 ▸; Richter *et al.*, 2014 ▸; Rothe & Skerra, 2018 ▸). Compared with conventional antibodies, Anticalins offer several advantages as they are significantly smaller (approximately 20 kDa compared with 150 kDa), which facilitates their cost-efficient production in prokaryotic expression systems such as *Escherichia coli*. Furthermore, Anticalins exhibit high thermal and chemical stability, which makes them resistant to harsh production and storage conditions. Finally, Anticalins lack an Ig Fc region, thus preventing undesired interactions with cells or the immune system.

In the context of pretargeted radioimmunotherapy and *in vivo* imaging, there is a growing demand for compact, robust and specific ligand-binding proteins to enhance tumor penetration, accelerate blood clearance and enable flexible exchange between radionuclides (*e.g.*^68^Ga/^90^Y, ^111^In/^177^Lu) while maintaining high affinity for the metal-chelate complexes (Boswell & Brechbiel, 2007 ▸; Burkett *et al.*, 2023 ▸; Goldenberg & Sharkey, 2006 ▸). In this context, Anticalins that have been developed for high affinity towards the rare-earth metal chelates Y^3+^·DTPA, Tb^3+^·DTPA and Lu^3+^·DTPA offer promising tools for *in vivo* diagnostics and radionuclide therapy (Eggenstein *et al.*, 2014 ▸; Kim *et al.*, 2009 ▸). Another recent application of these Anticalins relates to the PET-tracking of CAR T-cells in lymphoma models using an [^18^F]-labeled derivative of DTPA charged with a nonradioactive lanthanide metal ion (Morath *et al.*, 2025 ▸).

The initially selected Anticalin variants C26 (*K*_d_ ≃ 0.4 n*M* towards Y^3+^·DTPA) and L1 were subjected to *in vitro* affinity maturation, yielding the mutant CL31, which features a fourfold slower off-rate (>2 h dissociation half-life) and improved association kinetics towards several *M*^III^·DTPA complexes (Eggenstein *et al.*, 2014 ▸; Kim *et al.*, 2009 ▸). Further optimization involved four loop mutations, M44E, S86G, P87S and A145T, relative to CL31, resulting in the Anticalin CL31d, conferring superior thermostability and again better *M*^III^·DTPA affinity. Thus, the Anticalin CL31d offers a promising protein reagent for metal-complex binding in nuclear medicine, for example, as part of a bispecific fusion protein or when conjugated to an antibody, such as in the Mabcalin format (Luo *et al.*, 2026 ▸). In this regard, knowledge of the affinities towards DTPA complexes of various lanthanide and related main-group metal ions and a potential dependency on their ionic radii appears relevant. To this end, we here describe a systematic analysis of the binding properties of the Anticalin CL31d for various *M*^III^·DTPA complexes both at the functional and structural level.

In the context of applications in nuclear medicine, potential ion preferences are particularly relevant, encompassing a series of clinically established and emerging diagnostic and therapeutic radiometals. Among these are ^68^Ga^3+^ and ^43/44^Sc^3+^ for PET imaging (β^+^ emitters) as well as ^111^In^3+^ for SPECT imaging (γ emitter) (Boswell & Brechbiel, 2007 ▸). Furthermore, ^153^Sm^3+^, ^90^Y^3+^, ^161^Tb^3+^, ^165^Dy^3+^, ^177^Lu^3+^ and ^47^Sc^3+^ have been investigated as β^−^ emitters (Giese, 2018 ▸), while ^161^Tb^3+^ opens perspectives as an Auger electron emitter for targeted radiotherapy (Fricke *et al.*, 2026 ▸). Therefore, we extended the previous characterization of the binding activity of CL31d towards metal-chelate complexes to a panel of 11 different *M*^III^·DTPA complexes, thus covering this series of medically relevant trivalent metal ions.

## 2. Materials and methods

### 2.1. Preparation of *M*^III^·DTPA chelate complexes

An equimolar solution of YCl_3_·6H_2_O, TbCl_3_·6H_2_O, LuCl_3_·6H_2_O, GdCl_3_·6H_2_O, SmCl_3_·6H_2_O, HoCl_3_, InCl_3_, ScCl_3_ (all >99.99%; Sigma–Aldrich, Schnelldorf, Germany), BiCl_3_, GaCl_3_ (all >99.99%; Thermo Fisher Scientific, Darmstadt, Germany), DyCl_3_·*x*H_2_O, Ga(NO_3_)_3_·*x*H_2_O (all >99.99%; Carl Roth, Karlsruhe, Germany) and [(*R*)-2-amino-3-(4-aminophenyl)propyl]-*trans*-(*S*,*S*)-cyclohexane-1,2-diaminepentaacetic acid (*p*-NH_2_-BnCHX-A″-DTPA or, in brief, DTPA-NH_2_; Macrocyclics, Plano, Texas, USA) was mixed (at stoichiometric ratios stated in the text below) in 100 m*M* ammonium acetate pH 5.0 (Carl Roth) and incubated for 10 min at room temperature. The resulting *M*^III^·DTPA complexes were subsequently used for affinity measurements with the Anti­calin by fluorescence titration.

### 2.2. Soluble protein production and purification

The Anticalin CL31d (Eggenstein *et al.*, 2014 ▸) was produced in the periplasm of *E. coli* strain TG1/F^−^ (Kim *et al.*, 2009 ▸) using the expression vector pNGAL15 according to published procedures (Gebauer & Skerra, 2012 ▸). Briefly, the bacteria were cultured in 2 l LB medium supplemented with 100 mg l^−1^ ampicillin at 30°C and agitated in a shake flask at 150 rev min^−1^ until an OD_550_ ≃ 2.0 was reached. Recombinant gene expression was then induced by adding 200 µg l^−1^ anhydrotetracycline (aTc; Acros Organics, Geel, Belgium) for 3 h. The Anticalin was purified from the periplasmic cell extract via affinity chromatography using the Strep-tag II system (Schmidt & Skerra, 2007 ▸). Finally, preparative size-exclusion chromatography (SEC) on a Superdex 75 16/60 GL column (GE Healthcare, Freiburg, Germany) was performed with phosphate-buffered saline (PBS; 4 m*M* KH_2_PO_4_, 16 m*M* Na_2_HPO_4_, 115 m*M* NaCl pH 7.4) or with 10 m*M* Tris–HCl, 100 m*M* NaCl pH 8.0 as running buffers, respectively, for subsequent fluorescence titration or protein crystallization. Protein purity was verified by SDS–PAGE (Fling & Gregerson, 1986 ▸) and protein concentrations were determined by measuring the absorption at 280 nm using a calculated extinction coefficient of 23.045 *M*^−1^ cm^−1^ (Gasteiger *et al.*, 2003 ▸).

### 2.3. Fluorescence titration

The binding activity of the Anticalin CL31d towards different *M*^III^·DTPA complexes in solution was measured using a FluoroMax-3 spectrofluorimeter (HORIBA Jobin Yvon, Unterhaching, Germany) operated with the *FluorEssence* software version 3.1. A freshly purified 1 µ*M* protein solution in 2 ml PBS was placed in a quartz cuvette (10 mm path length; Hellma, Mühlheim, Germany) equipped with a tiny magnetic stirrer. The temperature was set to 25°C and, after reaching equilibrium, fluorescence of the protein Tyr/Trp residues was excited at 280 nm. The fluorescence intensity was recorded at 340 nm, corresponding to the emission maximum. A 100 µ*M* solution of the *M*^III^·DTPA complex in 100 m*M* ammonium acetate pH 5.0 was added stepwise, under stirring, up to a maximum volume of 40 µl (2% of the total sample volume), each time followed by 3 min incubation and measurement of the steady-state fluorescence. To calculate the *K*_d_ value, the data were normalized to an initial fluorescence intensity of 100% (corresponding to the plain protein solution, disregarding the subsequent dilution by the small ligand aliquots) and plotted against the total ligand concentration at each titration step. The data were then fitted via nonlinear least-squares regression based on the law of mass action for bimolecular complex formation with the *KaleidaGraph* software version 4.0 (Synergy, Reading, Pennsylvania, USA) using a published formula (Breustedt *et al.*, 2006 ▸).

### 2.4. Crystallization of CL31d with DTPA-NH_2_ in complex with different metal ions

The purified Anticalin CL31d was concentrated with a Vivaspin concentrator (10 kDa cutoff; Sartorius, Göttingen, Germany) and sterile-filtered with Costar Spin-X centrifuge units (0.45 µm; Sigma–Aldrich). For complex formation prior to crystallization, the corresponding solution of *M*^III^·DTPA-NH_2_ was added to the protein solution in a 1.3:1 molar ratio (metal-chelate ligand:protein) for lanthanide ions and in a 3:1 molar ratio for main-group metal ions. Prior to this, metal ions were mixed with DTPA-NH_2_ in a 10:1 molar ratio (metal:DTPA), except for Bi^3+^ and Dy^3+^, which were mixed with DTPA-NH_2_ in a 1:1 ratio. The final protein concentration was adjusted to 15–19 mg ml^−1^. Altogether, six different *M*^III^·DTPA-NH_2_–Anticalin complexes were crystallized (Table 1 ▸) using the hanging-drop vapor-diffusion method. The drops consisted of 1 µl *M*^III^·DTPA-NH_2_–Anticalin solution and 1 µl precipitant solution (for Lu^3+^, Tb^3+^, Sc^3+^, Bi^3+^ and Ga^3+^) or of 1.25 µl *M*^III^·DTPA-NH_2_–Anticalin and 1.25 µl precipitant solution (for Dy^3+^). The protein–ligand complexes were crystallized at 20°C using PEG 3350 and KH_2_PO_4_ as precipitants. Crystals grown at high PEG concentrations [34–36%(*w*/*v*)] were directly harvested from the droplets and immediately frozen in liquid nitrogen (for Bi^3+^, Lu^3+^ and Sc^3+^), whereas those from droplets with lower PEG concentrations [19–27%(*w*/*v*)] were first transferred into the respective precipitant buffer supplemented with 20%(*v*/*v*) glycerol (for Tb^3+^ and Dy^3+^) or 25%(*v*/*v*) glycerol (for Ga^3+^) prior to freezing (Supplementary Table S2).

### 2.5. Data collection and model building

Synchrotron X-ray diffraction data sets for the different protein crystals (Table 1 ▸) were collected on beamline P13 (for Lu^3+^, Tb^3+^, Sc^3+^, Bi^3+^ and Ga^3+^) of the PETRA III storage ring operated by EMBL (DESY, Hamburg, Germany) and on beamline X06SA (for Dy^3+^) of the Swiss Light Source (SLS; Paul Scherrer Institute, Villigen, Switzerland). The data were either processed with *XDS* and *XSCALE* (Kabsch, 2010 ▸; for Lu^3+^, Tb^3+^, Sc^3+^, Bi^3+^ and Ga^3+^) or with *MOSFLM* and *SCALA* from *CCP*4 (Agirre *et al.*, 2023 ▸; for Dy^3+^). Molecular replacement was performed with *Phaser* (Evans & McCoy, 2008 ▸) using the coordinate set of the Lcn2 variant CL31 with the bound chelate complex between Y^3+^ and a Tris adduct of *p*-SCN-Bn-CHX-A″-DTPA (PDB entry 4iax). The initial structure of the *M*^III^·DTPA-NH_2_ ligand was adopted from the same coordinate set. Atomic models were built with *Coot* (Emsley & Cowtan, 2004 ▸) and refined with *REFMAC*5 (from *CCP*4) in iterative cycles. Water molecules were added with *ARP*/*wARP* (Langer *et al.*, 2008 ▸). The resulting models were rectified using the *PDB-REDO* server (Joosten *et al.*, 2014 ▸) and validated using *PROCHECK* (Laskowski *et al.*, 1993 ▸) and the *MolProbity* server (Williams *et al.*, 2018 ▸). Protein–ligand interactions were analyzed with *PISA* (Krissinel & Henrick, 2007 ▸). Graphics and structural alignments were prepared with *PyMOL* (Schrödinger, New York, USA). The atomic coordinates and structure factors of the refined structures have been deposited in the Protein Data Bank (PDB), Research Collaboratory for Structural Bioinformatics (RCSB; Rutgers University, New Brunswick, New Jersey, USA) under accession codes 28xi (H_2_O·DTPA-NH_2_), 28xn (Lu^3+^·DTPA-NH_2_), 28xl (Tb^3+^·DTPA-NH_2_), 28xm (Sc^3+^·DTPA-NH_2_), 28xk(Bi^3+^·DTPA-NH_2_) and 28xj (Dy^3+^·DTPA-NH_2_).

## 3. Results and discussion

### 3.1. Investigation of the affinities between the Anticalin CL31d and different *M*^III^·DTPA-NH_2_ complexes by fluorescence titration

**Figure 1:**
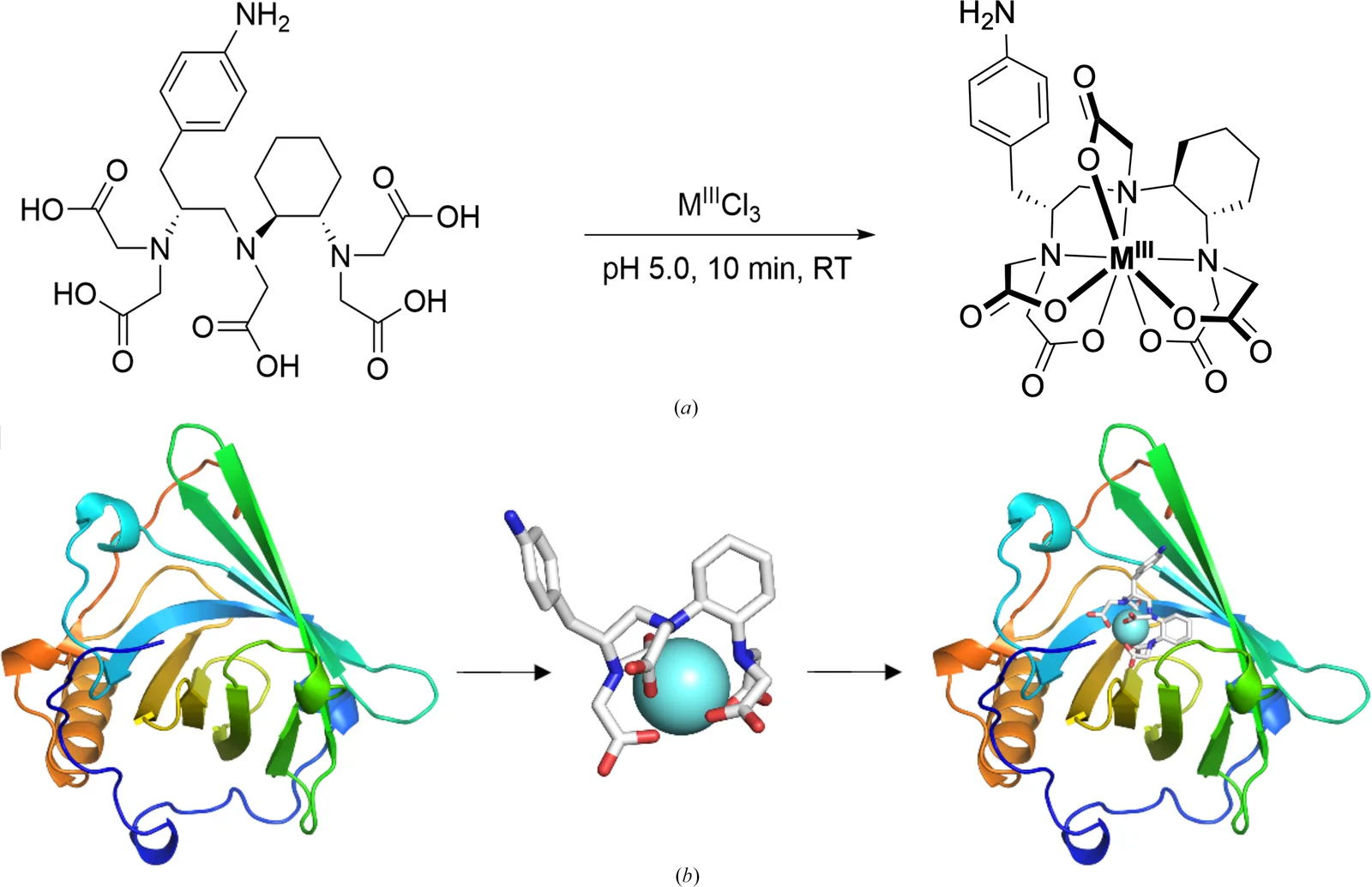
Structures of the metal-chelate complexes and their binding to the Anticalin. (*a*) Formation of an octadentate *M*^III^·DTPA-NH_2_ complex and its chemical configuration. (*b*) Binding of the metal chelate to the ligand pocket of the Anticalin CL31d at the open end of the β-barrel.

Previous work had demonstrated the selection and structural characterization of Anticalins that bind metal-chelate complexes formed between DTPA-NH_2_ and certain lanthanide ions (Kim *et al.*, 2009 ▸), such as Y^3+^, Tb^3+^ and Lu^3+^, whose radioisotopes are widely used in nuclear medicine for diagnostic imaging and targeted radiotherapy (Milenic *et al.*, 2004 ▸; Boswell & Brechbiel, 2007 ▸; Goldenberg & Sharkey, 2006 ▸; Han & Lee, 2026 ▸). Rational mutagenesis led to the improved Anticalin CL31d, showing high-affinity recognition of the hapten-like metal-chelate complex, with a dissociation constant (*K*_d_) of 4.6 n*M*, measured for Y^3+^·DTPA-NH_2_ via fluorescence titration, and of 0.5 n*M*, measured for a Y^3+^·DTPA-RNaseA conjugate via real-time surface plasmon resonance (SPR) spectroscopy (Eggenstein *et al.*, 2014 ▸). Building upon the previously described crystal structure of the parental variant CL31 with a bound Y^3+^·DTPA chelate derivative (PDB entry 4iax), the present investigation focused on the engineered mutant CL31d with bound chelate ligand DTPA-NH_2_ in complex with different trivalent metal ions (Fig. 1 ▸).

**Figure 2:**
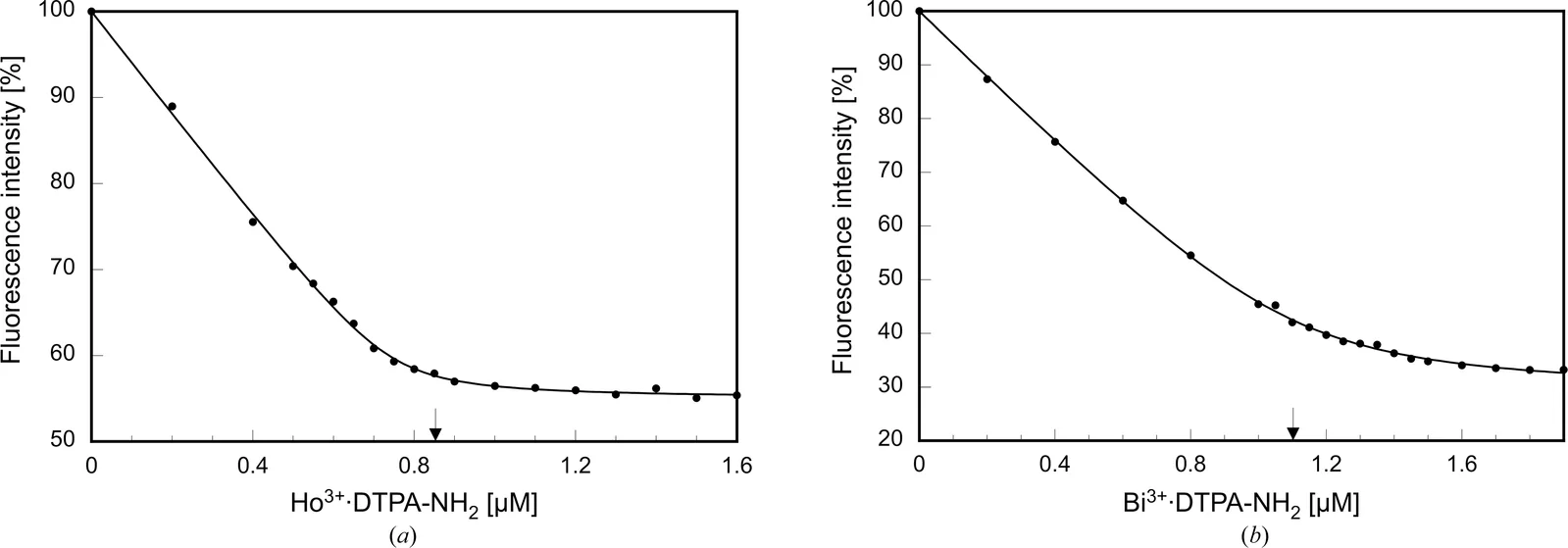
Exemplary fluorescence titration of the Anticalin CL31d with (*a*) Ho^3+^·DTPA-NH_2_ and (*b*) Bi^3+^·DTPA-NH_2_. The ligand solution was added stepwise to an approximately 1 µ*M* protein solution in PBS, and the protein Tyr/Trp fluorescence upon excitation at 280 nm was measured at 340 nm. Data were subjected to curve fitting according to the law of mass action for bimolecular complex formation. The equivalence point (0.85 or 1.10 µ*M*, respectively, resulting from the curve fit) is indicated by a small vertical arrow. Fluorescence titration of CL31d was also performed with DTPA-NH_2_ in complex with Sm^3+^, Gd^3+^, Tb^3+^, Dy^3+^, Y^3+^, Lu^3+^, In^3+^, Sc^3+^ and Ga^3+^, and without metal ion (for the resulting *K*_d_ values, see Table 2 ▸).

The Anticalin CL31d was produced in the soluble folded state, including one structural disulfide bridge (Eggenstein *et al.*, 2014 ▸; Schiefner & Skerra, 2015 ▸), in the periplasm of *E. coli* and purified via the Strep-tag II (Schmidt & Skerra, 2007 ▸), followed by size-exclusion chromatography (SEC). Its binding activity towards different *M*^III^·DTPA-NH_2_ chelate complexes in solution was analyzed via fluorescence titration using the ligand-quenching effect on the emission by Tyr/Trp side chains of the protein for read-out (Eggenstein *et al.*, 2014 ▸; Kim *et al.*, 2009 ▸). In this manner, 11 different *M*^III^ ions of the lanthanide series and adjacent main-group III and V elements, with ionic radii ranging from 0.62 to 1.03 Å, in complex with the DTPA-NH_2_ chelate ligand were investigated (Fig. 2 ▸, Table 2 ▸).

**Figure 3:**
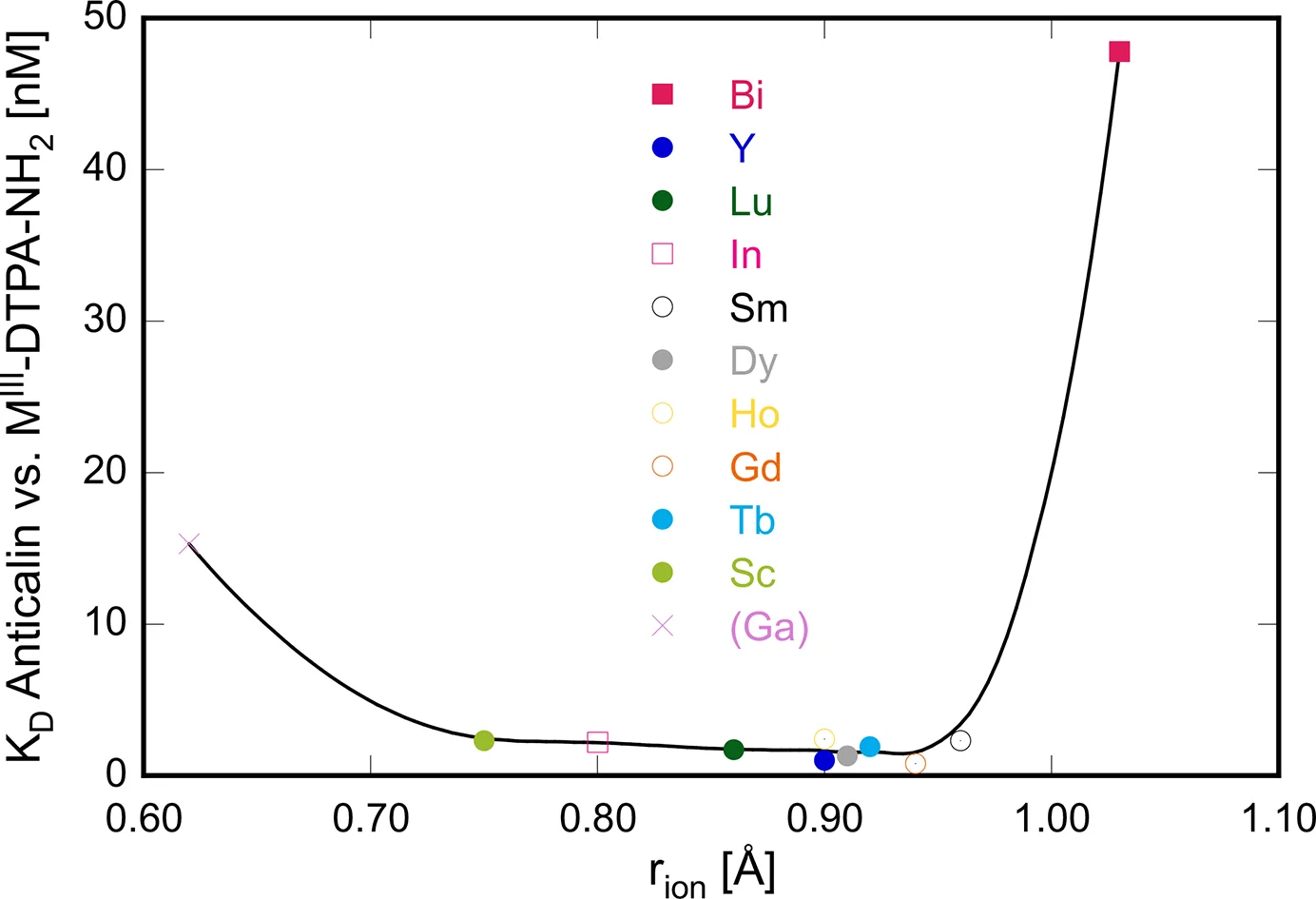
Affinities of the *M*^III^·DTPA-NH_2_ chelate complexes towards the Anti­calin CL31d plotted against the respective ionic radius of the metal (CN = 6). Spheres correspond to lanthanide elements, while squares represent main-group metals; filled symbols denote those metal ions whose complexes with an Anticalin were elucidated by X-ray crystallography. The curve fit of the data points from fluorescence titration measurements is based on a spline function.

According to the measured *K*_d_ values, CL31d exhibits strong and surprisingly uniform affinity, in the range 0.8–2.4 n*M*, towards the DTPA-NH_2_ chelator charged with different trivalent metal ions from the lanthanide series (Table 2 ▸, Fig. 3 ▸), in particular those with ionic radii close to 0.90 Å (0.75–0.96 Å), as reported for coordination number (CN) = 6 (Shannon, 1976 ▸). In contrast, fluorescence titration with DTPA-NH_2_ complexes of the main-group metals Ga^3+^ (*K*_d_ = 15.3 n*M*; *r*_ion_ = 0.62 Å) and Bi^3+^ (*K*_d_ = 47.8 n*M*; *r*_ion_ = 1.03 Å) indicated 6- to 60-fold lower affinities, also in relation to those main-group metals whose ionic radii are more similar to the lanthanides, such as Sc^3+^ (0.75 Å) and In^3+^ (0.80 Å). Regarding potential applications of the corresponding radionuclides in nuclear medicine, this suggests that any clinically useful metal ion with a radius around 0.90 Å may be employed as a DTPA-NH_2_ chelate complex with the Anti­calin CL31d. This would relate, for example, to the radioisotopes ^90^Y^3+^, ^177^Lu^3+^, ^161^Tb^3+^, ^47^Sc^3+^, ^153^Sm^3+^, ^165^Dy^3+^ and ^166^Ho^3+^ for therapy or to ^111^In^3+^, ^43/44^Sc^3+^ and ^153^Gd^3+^ for *in vivo* diagnostics (Boswell & Brechbiel, 2007 ▸; Giese, 2018 ▸; Han & Lee, 2026 ▸; Sadler *et al.*, 2022 ▸; Van Laere *et al.*, 2024 ▸).

In a preceding study on the 2D12.5 Fab fragment, Corneillie and coworkers reported a parabolic affinity relationship for the binding activity towards DOTA–lanthanide complexes (Corneillie, Whetstone *et al.*, 2003 ▸), with a peak for metal ions with an ionic radius (*r*_ion_) of approximately 1.115 Å (Gd^3+^, 1.107 Å; Eu^3+^, 1.120 Å) described for CN = 9 (corresponding to 0.944 Å for Gd^3+^ at CN = 6; see Table 2 ▸). Their findings indicated that smaller ions (*e.g.* Lu^3+^) contract the complex with the macrocycle, while larger ones (*e.g.* La^3+^) expand it. This likely leads to a distortion of the ligand geometry by displacing hydrophobic methylene groups from the binding pocket of the Fab and thereby increasing the free-energy penalty (ΔΔ*G*; Corneillie, Fisher *et al.*, 2003 ▸).

**Figure 4:**
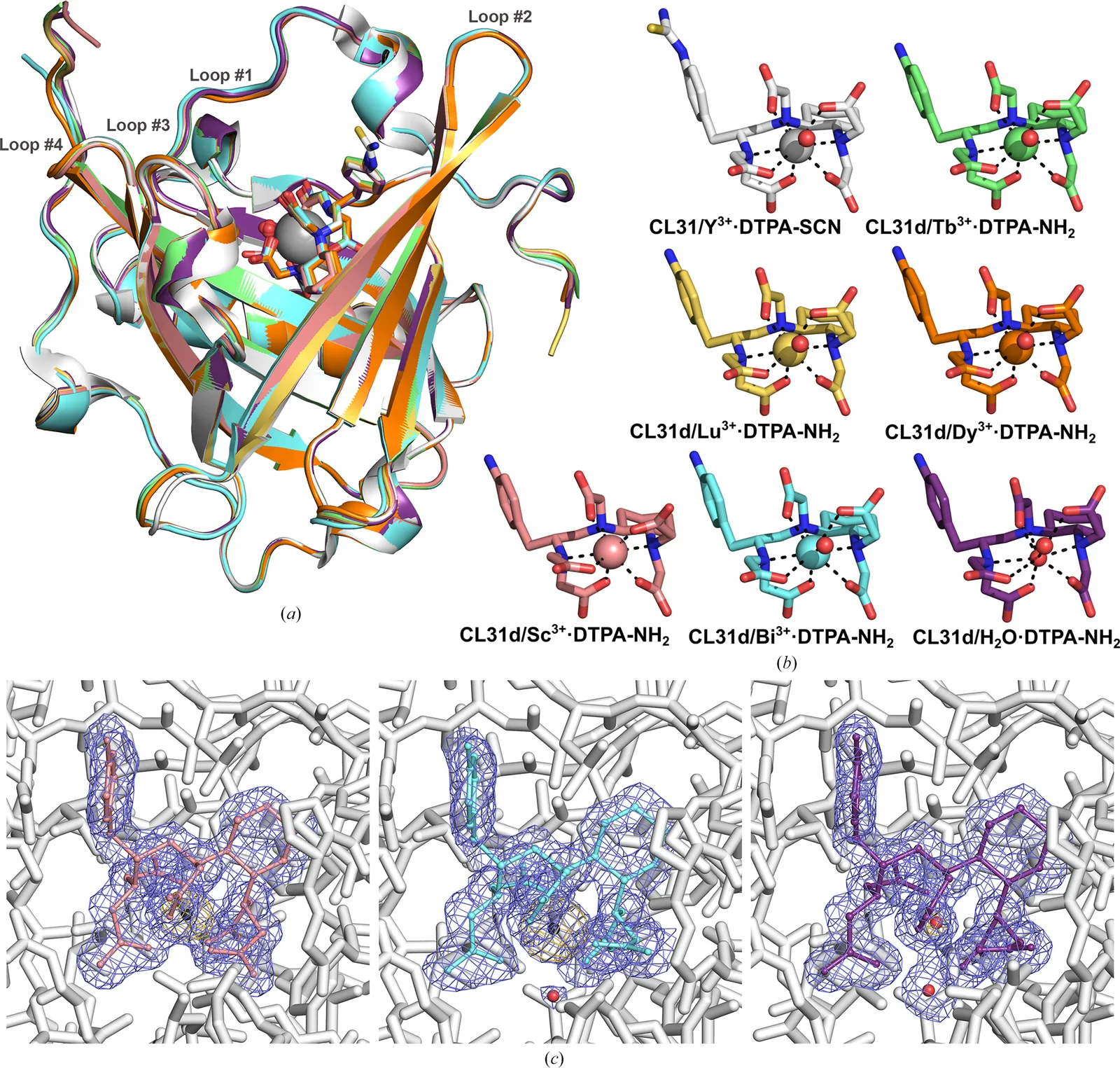
X-ray structural analysis of different* M*^III^·DTPA-NH_2_ chelate complexes bound to the Anticalin CL31d. (*a*) Superposition of the different CL31d structures with bound DTPA-NH_2_ chelate complexes of Bi^3+^ (cyan C atoms), Tb^3+^ (green), Dy^3+^ (orange), Lu^3+^ (yellow), Sc^3+^ (salmon) and the previously published Y^3+^·DTPA-SCN complex (white; PDB entry 4iax), as well as the metal-free DTPA-NH_2_ (purple; from co-crystallization with Ga^3+^) using a set of 58 structurally conserved C^α^ atoms of the lipocalin scaffold. (*b*) Individual *M*^III^·DTPA-NH_2_ complexes from the co-crystal structures with the Anticalin CL31d or CL31 together with the metal-coordinating water molecule (dashed lines indicate the eightfold or ninefold metal coordination). (*c*) Exemplary 2*F*_o_ − *F*_c_ electron densities for the DTPA-NH_2_ complexes of Sc^3+^ (left) and Bi^3+^ (center) in comparison with the metal-free chelate ligand (obtained in the presence of Ga^3+^), which harbors a water molecule at the position of the central ion (right). The 2*F*_o_ − *F*_c_ electron densities are contoured at 1σ in blue for DTPA-NH_2_ and additonally at 3σ in yellow-orange for the central ion or water molecule.

In comparison, we observed a surprisingly flat affinity profile of the Anticalin CL31d for DTPA-NH_2_ chelate complexes with trivalent metal ions with ionic radii in the range from 0.75 to 0.96 Å, which suggests a more relaxed dependence (Fig. 3 ▸). In fact, the branched linear chemical configuration of the DTPA chelator and its resulting compact eight-coordinated complex structures, with five carboxylate O atoms and three N atoms as donors and, optionally, one additional H_2_O molecule as a ligand, appears to be more tolerant towards distortions caused by the size of the central ion, resulting in minimal geometric variation (Fig. 4 ▸).

Of note, for analysis across the range of metal ions investigated in the present study (Fig. 3 ▸) we used *r*_ion_ values reported for CN = 6 (Shannon, 1976 ▸), since non-lanthanide metal ions (in particular In^3+^, Sc^3+^ and Ga^3+^) lack standardized values for CN = 9, as they predominantly coordinate 6–8 ligand donors and rarely adopt ninefold coordination (Lau *et al.*, 2006 ▸; Meehan *et al.*, 1999 ▸; Shannon, 1976 ▸). Nevertheless, the relative trend in changes of *r*_ion_ between the different elements is similar, regardless of the specific CN applied (see Table 2 ▸). Indeed, when plotting the affinities between the Anticalin CL31d and the different *M*^III^·DTPA-NH_2_ chelate complexes against the ionic radii reported for CN = 9, to the extent available, a nearly identical curve was obtained (not shown).

### 3.2. Structural analysis of *M*^III^·DTPA-NH_2_ chelate complexes bound to the Anticalin CL31d and influence of the central ion on the geometry

To obtain detailed insight into how the central ion of lanthanides and chemically related metals influences the configuration of the chelate complex and its interactions within the engineered lipocalin pocket, we determined X-ray structures of CL31d co-crystallized with the DTPA-NH_2_ complexes of Bi^3+^, Tb^3+^, Dy^3+^, Lu^3+^, Sc^3+^ and Ga^3+^ at high resolution (1.5–1.8 Å; Table 1 ▸; Fig. 4 ▸). These metals span ionic radii from 0.62 to 1.03 Å (CN = 6) and include clinically relevant ions such as Tb^3+^ and Lu^3+^, whose radioisotopes (^161^Tb^3+^ and ^177^Lu^3+^, respectively) have found increasing use in targeted radionuclide therapy (Van Laere *et al.*, 2024 ▸; Trejtnar *et al.*, 2025 ▸). Furthermore, the main-group metal ions Bi^3+^ and Ga^3+^, with ^213^Bi^3+^ of relevance as a therapeutic α emitter (Nawar *et al.*, 2025 ▸) and ^68^Ga^3+^ as a β^+^ emitter for PET imaging (Morath *et al.*, 2023 ▸), were chosen due to their significantly larger/smaller ionic radii as well as their considerably lower measured affinities towards the Anticalin when applied in complex with DTPA-NH_2_ (Table 2 ▸).

**Figure 5:**
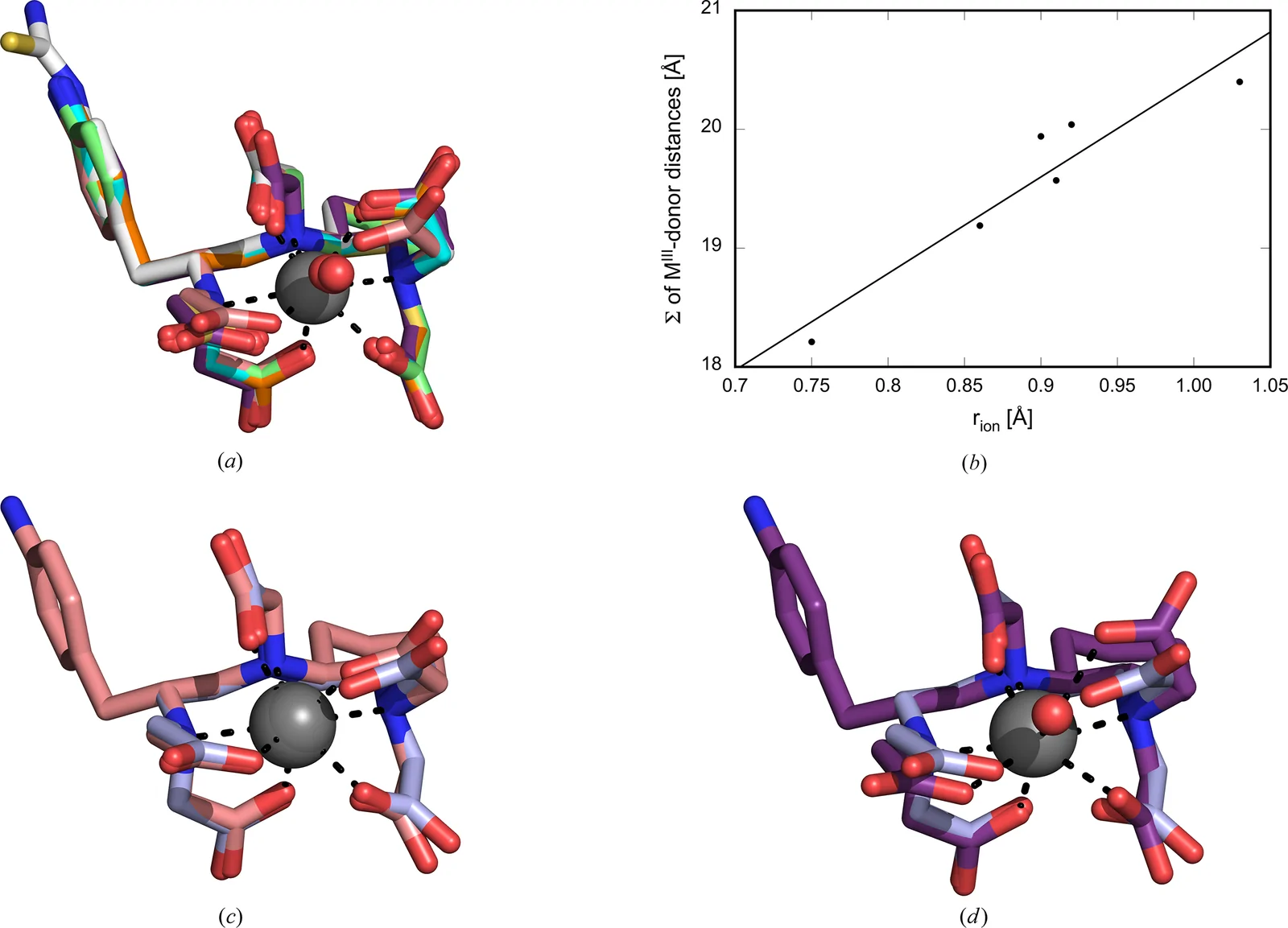
Correlation between the radius of the central ion and the overall geometry of the Anticalin-bound metal-chelate complex. (*a*) Superposition of the six different *M*^III^·DTPA-NH_2_ complexes via the central ion to illustrate the minute structural influence of the ionic radius: Bi^3+^·DTPA-NH_2_ (cyan C atoms), Tb^3+^·DTPA-NH_2_ (green), Dy^3+^·DTPA-NH_2_ (orange), Y^3+^·DTPA-NH_2_ (white), Lu^3+^·DTPA-NH_2_ (yellow) and uncomplexed DTPA (purple). (*b*) Sum of the metal–ligand distances to eight donor atoms (5 O + 3 N) plotted against the ionic radii (CN = 6). (*c*, *d*) Superposition of the Sc^3+^·DTPA-NH_2_ complex (salmon) and of the empty DTPA-NH_2_ chelator (purple; harboring a central water molecule, which is obscured here, and one peripherally associated water molecule), respectively, with the published In^3+^·DTPA complex (light blue) from the Cambridge Structural Database (CSD entry MOQVOD). Contacts with the coordinating N and O atoms and the water molecule are indicated by dashed black lines.

Across all six structures, both the binding protein and the metal-chelate ligand accommodated in its pocket revealed remarkably conserved conformations. Superposition of the β-barrel core and non-engineered loops using 58 structurally conserved C^α^ atoms (Achatz *et al.*, 2022 ▸) yielded an average root-mean-square deviation (r.m.s.d.) of only 0.095 Å relative to the parental CL31–Y·DTPA-SCN structure (PDB entry 4iax), which moreover deviates by four amino-acid substitutions (M44E/S86G/P87S/A145T; Eggenstein *et al.*, 2014 ▸). Similarly, individual superposition of the different DTPA-NH_2_ chelate ligands (without the central ion) led to an r.m.s.d. of 0.14 Å, on average, for 39 atoms (Fig. 5 ▸). In all complexes, the trivalent metal ion was seen to be encapsulated by the multidentate DTPA chelator, whose nitrogen and carboxylate donor groups form the characteristic *M*^III^·DTPA core and also engage in a network of hydrogen bonds and salt bridges with polar and charged side chains that line the ligand pocket of the protein.

Similar to the crystal structure of the parental CL31–Y·DTPA-SCN complex, the Me^III^·DTPA core group is bound among the side chains of residues Gln33, Arg36, Thr52 and Gln54 in loop 1, Val66, Ala68, Arg70, Glu77, Tyr78 and Leu79 in loop 2, Tyr106 and Phe123 in loop 3 as well as Ser136 in loop 4, which together form the four hypervariable loops of the lipocalin scaffold (Achatz *et al.*, 2022 ▸; Skerra, 2000 ▸). The shorter *p*-NH_2_-benzyl linker, compared with the Tris-quenched Y^3+^·DTPA-SCN employed in the preceding crystallo­graphic study (Eggenstein *et al.*, 2014 ▸), consistently docks against the ridge formed by the side chains of Leu79 as well as Arg70 and Glu77. Likewise, the chiral cyclohexylene group was observed to pack against the hydrophobic residues Val66, Ala68, Leu79, Met81 and Phe83 in all complexes of CL31d-bound *M*^III^·DTPA-NH_2_, including the previously elucidated CL31–Y^3+^·DTPA-SCN complex. The chelate arms of the DTPA moiety maintain identical hydrogen bonds to Gln33 (O^ɛ1^, N^ɛ2^), Thr52 (O^γ^), Gln54 (N^ɛ2^), Tyr106 (OH) and Ser136 (O^γ^) at distances of 2.7–3.5 Å.

Regarding the metal ion coordination geometry, all lanthanide complexes exhibit uniform ninefold coordination by the following donor groups/atoms: five (deprotonated) carboxylate O atoms, three tertiary N atoms and one bound water molecule. Notably, in the co-crystal structure with Sc^3+^, which represents a smaller main-group ion (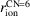 = 0.75 Å) two rows above La^3+^ in the periodic table of the elements, the water ligand is missing (Fig. 5 ▸*c*), resulting in an eightfold coordination with a distorted Archimedian antiprismatic configuration, similar to that described in the seminal crystallographic analysis of the Na_2_In(DTPA)·7H_2_O salt (Maecke *et al.*, 1989 ▸). Thus, it appears that all metal ions larger than Sc^3+^ and In^3+^ (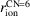 = 0.80 Å) investigated here, in particular those belonging to the lanthanide series, exhibit an expanded coordination sphere where a water molecule from the solvent occupies the ninth position. Obviously, this molecular behavior is independent of the precise electronic configuration of the central metal ion, as it is observed for four different lanthanides and even for the main-group metal ion Bi^3+^ (which has a fully occupied shell of *d* and *f* electrons).

Unexpectedly, in the crystallographic analysis performed for the CL31d–*M*^III^·DTPA-NH_2_ complex in the presence of Ga^3+^, which shows an even smaller ionic radius (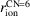 = 0.62 Å) than Sc^3+^ (see Table 2 ▸), no bound metal ion was detectable; instead, the electron-density map revealed a spherical signal characteristic of a discrete water molecule (Fig. 4 ▸*b* and 4 ▸*c*). This situation did not change in repeated co-crystallization trials using a threefold excess of DTPA-NH_2_ and up to a tenfold excess of GaCl_3_ relative to the Anticalin protein. Indeed, confirmatory fluorescence titration measurements underlined an incomplete chelation: use of ‘Ga^3+^·DTPA-NH_2_’ as a ligand indicated a *K*_d_ value of 15.3 ± 1.9 n*M*, while a fluorescence titration of CL31d with empty DTPA-NH_2_ (without metal ion added) resulted in *K*_d_ = 12.1 ± 0.9 n*M*, both values being statistically indistinguishable (Supplementary Fig. S1). This finding aligns with the chemical properties known for Ga^3+^: it forms weak, kinetically labile complexes with chemically related cyclic chelators, such as DOTA (log*K* ≃ 22–26, compared with 27.5 for Lu^3+^; Kubícek *et al.*, 2010 ▸), but it favors a (distorted) octahedral (hexadentate) coordination over the octadentate complex formation typical for DTPA, due to its small radius and pronounced tendency to hydrolysis.

Indeed, a previous ^13^C-NMR analysis of the Ga(DTPA)^2−^ complex indicated that while this complex appeared to be stable in neutral solution, the central Ga^3+^ ion adopts its preferred octahedral coordination, with only three COO^−^ groups engaged (together with the three N atoms of DTPA) and two COOH groups in an uncomplexed state (Maecke *et al.*, 1989 ▸). Obviously, the resulting peculiar geometry of this chelate complex is not compatible with binding to the ligand pocket of the Anticalin CL31d. On the other hand, from the perspective of the engineered lipocalin, it is astonishing that this artificial binding protein appears to recognize the empty chelator with relatively strong affinity and in a conformation that is amenable to tight metal-ion complexation (Figs. 4 ▸*b* and 4 ▸*c*). This might be an effect of the (chiral) cyclohexylene moiety introduced into the backbone of the otherwise acyclic DTPA chelator employed here, which is known to favor a complexation-competent conformation, leading to kinetic inertness of Y^3+^ (and related metal ion) complexes in comparison with plain DTPA (McMurry *et al.*, 1998 ▸). In fact, a direct structural comparison of the protein-bound Sc^3+^·DTPA-NH_2_ complex solved in the present study with the previously published Na_2_In(DTPA)·7H_2_O salt (Fig. 5 ▸*c*) proves that the rigidification of the DTPA backbone by the cyclohexylene group does not affect the octadentate complex geometry.

### 3.3. Detailed analysis of the ligand geometry in the CL31d-bound *M*^III^·DTPA-NH_2_ complexes

Mutual comparison of the different Anticalin-bound metal-chelate complexes did not reveal noticeable changes due to the identity or ionic radius of the individual metals investigated. In fact, a superposition of the DTPA-NH_2_ chelator across all complexes yielded very small r.m.s.d. values of 0.13–0.39 Å for a total of 39 equivalent atom pairs (Fig. 5 ▸*a*). The Sc^3+^·DTPA-NH_2_ and H_2_O·DTPA-NH_2_ structures showed slightly larger deviations (0.39 and 0.26 Å, respectively), which were mostly attributable to contracted carboxylate arms (Supplementary Table S1) and the missing additional water ligand or central ion, respectively. Nevertheless, the orientation of the DTPA-NH_2_ molecule within the Anticalin pocket remained essentially invariant, as shown by conserved protein side-chain positions and hydrogen-bonding patterns involving residues Ser87, Asp89 and Tyr132 (see above).

For further analysis, we quantified the individual metal–ligand distances to the eight coordinating atoms of the DTPA-NH_2_ chelator (5 O + 3 N). Again, the resulting pattern (Supplementary Table S1) revealed only minute differences, also when including the additional water ligand. This means that the overall geometry of the *M*^III^·DTPA-NH_2_ complexes is mostly determined by the chemical constitution of the octadentate chelator, which acts as a rigid cage, rather than by the ionic radius of the central metal ion. However, when adding up the individual metal–donor distances, the small individual changes accumulated and a linear scaling with the ionic radius became apparent (Fig. 5 ▸*b*), with a Pearson correlation coefficient *R* = 0.948. A similar picture appeared when including the water ligand that is present in the complexes (except for Sc^3+^; not shown).

Interestingly, only for the largest ion tested, Bi^3+^ (1.03 Å), were structural deviations in the Anticalin-bound metal-chelate complex detectable. The average metal–donor distances were elongated by 0.12 Å (see Supplementary Table S1), while the water-mediated hydrogen-bonding pattern around the DTPA chelate remained essentially conserved. The position of a single bridging water molecule between the carboxylate oxygens O2 and O5 differed by about 0.1 Å upon superposition with the H_2_O·DTPA-NH_2_ complex. These significant structural perturbations provide a rationale for the approximately 60-fold weaker affinity (*K*_d_ = 47.8 n*M*) of CL31d towards the DTPA-NH_2_ complex with the main-group ion Bi^3+^ relative to the lanthanide ions. At the other end of the series of metal ions with differing sizes, the cumulated metal–donor distances revealed the smallest value for the main-group metal ion Sc^3+^ (18.21 Å versus 19.19 Å for Lu^3+^, the smallest lanthanide investigated). Nevertheless, Sc^3+^·DTPA-NH_2_ retained a high affinity towards the Anticalin (*K*_d_ = 2.3 n*M*), despite the missing additional water ligand, thus demonstrating a broad tolerance of its binding pocket towards variation in ionic radius as long as the gross geometry of the chelate complex is retained.

The essentially linear correlation between the sum of the metal–donor distances and the ionic radii of the *M*^III^·DTPA-NH_2_ chelate complexes (Fig. 5 ▸*b*, Supplementary Table S1) is in agreement with the conserved molecular recognition by the Anticalin CL31d, revealing a broad plateau in the measured affinities for 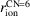 values 0.75–0.95 Å (Fig. 3 ▸). This contrasts with the binding properties described for antibodies directed against metal-chelate complexes, which typically display either a parabolic affinity pattern depending on the ionic radius or more extreme metal specificity. For instance, the 2D12.5 Fab exhibits moderate cross-reactivity among the series of lanthanide–DOTA complexes (optimal near 1.12 Å, CN = 9). In this case, smaller ions contract while larger ions expand the cyclic chelator, leading to steric strain with the acetate side chains (r.m.s.d. = 0.45 Å between Y^3+^·DOTA and Gd^3+^·DOTA; PDB entries 1nc2 and 1nc4; Corneillie, Fisher *et al.*, 2003 ▸). Conversely, the MAb CHA255 directed against In^3+^·benzyl-EDTA shows a >10^4^-fold preference for the complex with In^3+^ compared with other metal ions with similar radius, such as Fe^3+^ and Cd^2+^ (Love *et al.*, 1993 ▸). This is likely due to the unique axial coordination of In^3+^ by the N^ɛ^ atom of His H95 in the heavy-chain variable domain (N^ɛ^–In, 2.4 Å; PDB entry 1ind), which is lost with other metal ions because the chelate adopts a slightly different conformation that shields the central ion from interaction with this His side chain (Delehanty *et al.*, 2003 ▸). Similarly, the MAb 2C12 recognizes Pb^2+^·CHX-A′′-DTPA with 20–40 000-fold selectivity over different radius-matched metal ions (Khosraviani *et al.*, 2000 ▸).

## 4. Conclusions

This study demonstrates that the engineered Anticalin CL31d exhibits robust binding of *M*^III^·DTPA-NH_2_ chelate complexes across a series of trivalent metal ions spanning ionic radii of 0.75–1.03 Å with high affinities throughout (*K*_d_ = 0.8–2.4 n*M*). The mode of molecular recognition is surprisingly tolerant towards central ions with varying size and *d*/*f*-electron configuration, covering clinically relevant lanthanides with *r*_ion_ ≃ 0.90 Å, such as Y^3+^, Lu^3+^, Tb^3+^ and Dy^3+^, as well as In^3+^ and Sc^3+^. Only the DTPA complex of Bi^3+^, with a rather large *r*_ion_ = 1.03 Å, shows significantly weaker binding, whereas that of Ga^3+^, with an extremely small *r*_ion_ = 0.62 Å, assumes different chelate geometry and, thus, is no longer recognized by the Anticalin.

High-resolution crystal structures (1.5–1.8 Å) reveal nearly invariant geometries for the *M*^III^·DTPA-NH_2_ chelate complexes when tightly bound by the Anticalin, which contrasts with all antibodies directed against metal-chelate complexes that have been described so far, in particular those involving the cyclic DOTA chelator. In this context, the partially flexible backbone of the acyclic chelate ligand CHX-A″-DTPA, with the exception of the cyclohexylene moiety, enables chelate presentation of metal ions with radii across a considerable range while preserving molecular recognition by the Anticalin with low-nanomolar affinity.

These findings establish the Anticalin CL31d as a versatile non-immunoglobulin protein module for *in vivo* imaging, as recently demonstrated for the membrane-anchored DTPA receptor (Morath *et al.*, 2025 ▸) and, potentially, for pretargeted radionuclide delivery in the future (Goldenberg *et al.*, 2012 ▸). Generally, in the area of theranostics (Burkett *et al.*, 2023 ▸) the flexible exchange of radioisotopes, for example ^90^Y^3+^, ^177^Lu^3+^ and ^161^Tb^3+^ for tumor therapy or ^111^In, ^43^Sc^3+^ and ^68^Ga^3+^ for *in vivo* diagnostics, offers a crucial advantage. Apart from the low nanomolar avidity, the small size (∼20 kDa) of the Anticalin and its constitution as a robust single-chain protein render it beneficial for the construction of fusion proteins, either to directly label cells at their surface (Morath *et al.*, 2025 ▸) or to generate bispecific antibodies such as the Mabcalin format (Luo *et al.*, 2026 ▸; Wachter *et al.*, 2023 ▸). Future work should validate the application of radiolabeled chelates *in vivo* and explore different linker modalities to broaden the utility of the Anticalin CL31d for engineered protein theranostics.

## Supplementary Material

PDB reference: engineered human lipocalin 2 (CL31d), complex with H_2_O-DTPA, 28xi

PDB reference: complex with Dy-DTPA, 28xj

PDB reference: complex with Bi-DTPA, 28xk

PDB reference: complex with Tb-DTPA, 28xl

PDB reference: complex with Sc-DTPA, 28xm

PDB reference: complex with Lu-DTPA, 28xn

Supporting Information: Figure S1 and Tables S1 and S2. DOI: 10.1107/S2053230X26007624/rl5209sup1.pdf

## Figures and Tables

**Table 1:** X-ray data-collection and refinement statistics for CL31d–*M*^III^·DTPA-NH_2_ complexes Values in parentheses are for the highest resolution shell.

	Lu^3+^·DTPA-NH_2_	Dy^3+^·DTPA-NH_2_	Tb^3+^·DTPA-NH_2_	Sc^3+^·DTPA-NH_2_	Bi^3+^·DTPA-NH_2_	H_2_O·DTPA-NH_2_
Crystal data						
Space group	*P*4_1_2_1_2 [No. 92]	*P*4_1_2_1_2 [No. 92]	*P*4_1_2_1_2 [No. 92]	*P*4_1_2_1_2 [No. 92]	*P*4_1_2_1_2 [No. 92]	*P*4_1_2_1_2 [No. 92]
*a*, *b*, *c* (Å)	60.1, 60.1, 110.4	61.4, 61.4, 118.0	60.3, 60.3, 110.7	59.6, 59.6, 110.1	60.2, 60.2, 111.1	60.1, 60.1, 110.7
α, β, γ (°)	90, 90, 90	90, 90, 90	90, 90, 90	90, 90, 90	90, 90, 90	90, 90, 90
Molecules per asymmetric unit	1	1	1	1	1	1
Data collection						
Wavelength (Å)	0.97630	1.00001	0.97630	0.97620	0.97630	0.97620
Resolution range (Å)	60.07–1.45	61.25–1.80	110.70–1.60	42.14–1.60	55.56–1.60	60.10–1.50
〈*I*/σ(*I*)〉	9.0 (2.0)	4.3 (1.2)	14.0 (2.1)	17.7 (2.1)	14.6 (2.0)	25.8 (2.1)
*R*_meas_ (%)	8.5 (71.5)	10.8 (67.0)	6.4 (92.0)	4.0 (79.8)	5.3 (72.1)	3.2 (77.5)
Unique reflections	36362	21548	26313	26141	26684	32903
Multiplicity	3.6 (3.7)	12.6 (12.6)	5.9 (6.0)	3.6 (3.7)	3.7 (3.6)	4.4 (4.5)
Completeness (%)	99.0 (99.5)	99.9 (100.0)	94.6 (95.2)	96.7 (98.2)	96.0 (98.9)	98.7 (98.8)
Refinement						
*R*_cryst_/*R*_free_† (%)	17.0/19.6	19.2/22.7	17.4/20.1	18.3/21.8	17.4/20.1	17.5/20.3
Protein atoms	1425	1406	1418	1436	1402	1419
Ligand atoms	39	39	45 [+6 in glycerol]	39	39	39
Ions	2 [Lu^3+^, K^+^]	2 [Dy^3+^, K^+^]	2 [Tb^3+^, K^+^]	2 [Sc^3+^, K^+^]	4 [Bi^3+^, 2 K^+^, 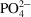 ]	1 [K^+^]
Solvent atoms	120	95	107	133	147	131 [+1 in DTPA]
Average *B* factor (Å^2^)	29.3	35.2	31.4	30.9	28.0	26.2
Geometry						
R.m.s.d., bond lengths (Å)	0.008	0.009	0.009	0.010	0.010	0.011
R.m.s.d., angles (°)	1.479	1.659	1.661	1.734	1.732	1.923
Ramachandran analysis						
Core (%)	92.8	92.0	92.1	90.9	92.6	92.8
Allowed (%)	5.3	6.0	5.9	6.5	5.4	5.9
Generously allowed (%)	0.7	0.7	0.7	0.6	0.7	0.0
Disallowed (%)	1.3	1.3	1.3	1.9	1.3	1.3

† *R*_free_ is *R*_cryst_ for 5% of the reflections that were randomly selected and excluded from refinement (Brünger, 1997 ▸).

**Table 2:** *K*_d_ values measured via fluorescence titration for the Anticalin CL31d with different metal ions in complex with DTPA-NH_2_ as a function of their ionic radii (depending on the coordination number, CN = 6–9; Shannon, 1976 ▸)

Central ion	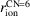 (Å)	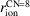 (Å)	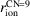 (Å)	*K*_d_ (n*M*)
Bi^3+^	1.03	1.17	—	47.8 ± 1.8
Sm^3+^	0.96	—	1.13	2.3 ± 0.8
Gd^3+^	0.94	—	1.11	0.8 ± 0.4
Tb^3+^	0.92	—	1.09	1.9 ± 0.7
Dy^3+^	0.91	—	1.08	1.3 ± 0.5
Y^3+^	0.90	—	1.07	1.0 ± 0.3
Ho^3+^	0.90	—	1.07	2.4 ± 1.0
Lu^3+^	0.86	—	1.03	1.7 ± 0.7
In^3+^	0.80	0.92	—	2.2 ± 0.9
Sc^3+^	0.75	0.87	—	2.3 ± 1.1
Ga^3+^	0.62	1.05	—	15.3 ± 1.9
(H_2_O)	—	—	—	(12.1 ± 0.9)
