# Supplementary material for: Diverse DTPA-chelated lanthanides with relevance for nuclear medicine bound to an engineered lipocalin reveal conserved ligand geometry
Source: Acta Crystallogr F Struct Biol Commun. 2026 Aug 17;82(Pt 9):301–11. doi: 10.1107/S2053230X26007624 (PMC13523712; doi:10.1107/S2053230X26007624)
Supplement: Supplementary file 1 [file f-82-00301-sup1.pdf]

Diverse DTPA-chelated lanthanides with relevance for nuclear medicine bound to an engineered lipocalin reveal conserved ligand geometry

Alina Mirwald, Andreas Eichinger, Arne Skerra*

Chair of Biological Chemistry, School of Life Sciences, Technical University of Munich, 85354 Freising, Germany

Supplementary Information

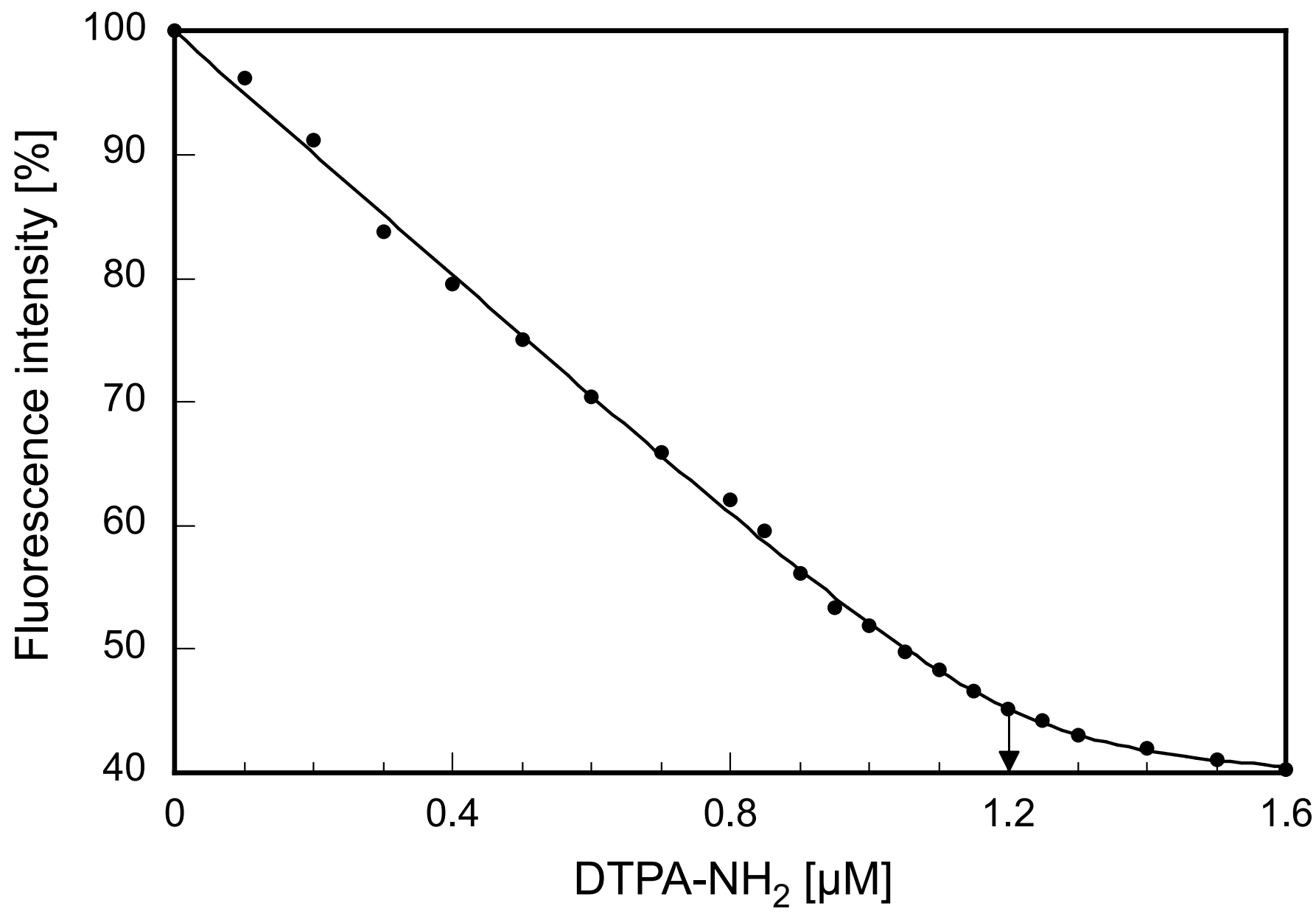

Figure S1. Fluorescence titration of the Anticalin CL31d with uncomplexed DTPA-NH_2 (no addition of a metal ion). The ligand solution was added stepwise to an approximately 1 µM protein solution in PBS, and the protein Tyr/Trp fluorescence was measured at 340 nm upon excitation at 280 nm. Data were subjected to curve fitting according to the Law of Mass Action for bimolecular complex formation. The K_D value was determined from the average of 3 fluorescence titrations (K_D = 12.0 ± 0.9 nM; N=3). The equivalence point (1.2 µM, resulting from the curve fit) is indicated by a small vertical arrow.

Table S1. Distances of DTPA donor atoms to the central ion [Å] in the M^{III}·DTPA-NH_2 complexes bound to the Anticalins CL31 or CL31d.

Donor atom	Bi^{3+}	Tb^{3+}	Dy^{3+}	Y^{3+}	Lu^{3+}	Sc^{3+}	H_2O
**O2**	2.63	2.44	2.48	2.48	2.34	2.19	2.89
**O4**	2.40	2.31	2.37	2.37	2.31	2.12	2.11
**O6**	2.42	2.38	2.33	2.34	2.24	2.19	2.19
**O8**	2.59	2.56	2.12	2.37	2.30	2.25	2.38
**O10**	2.63	2.48	2.39	2.46	2.29	2.13	3.02
**N1**	2.63	2.68	2.66	2.66	2.64	2.49	2.58
**N2**	2.52	2.57	2.59	2.61	2.51	2.40	2.30
**N3**	2.58	2.62	2.63	2.65	2.56	2.44	2.42
**Σ of distances**	20.40	20.04	19.57	19.94	19.19	18.21	19.89
**Water ligand**	2.81	2.43	2.44	2.41	2.45	–	2.92
**Σ including water**	23.21	22.47	22.01	22.35	21.64	–	22.81
$\boldsymbol{r_{\mathrm{ion}}^{\mathrm{CN=6}}}$	1.03	0.92	0.91	0.90	0.86	0.75	–

Table S2. Crystallization conditions of the CL31d/M^{III}·DTPA-NH_2 complexes.

Lu^{III}	36 % (w/v) PEG3350, 400 mM KH_2PO_4 pH 4.1
Dy^{III}	19 % (w/v) PEG3350, 400 mM KH_2PO_4 pH 4.1
Tb^{III}	20 % (w/v) PEG3350, 400 mM KH_2PO_4 pH 4.1
Sc^{III}	36 % (w/v) PEG3350, 400 mM KH_2PO_4 pH 4.1
Bi^{III}	34 % (w/v) PEG3350, 400 mM KH_2PO_4 pH 4.1
(Ga^{III})	27 % (w/v) PEG3350, 400 mM KH_2PO_4 pH 4.1
